# Anti-Osteoporotic Effects of the Herbal Mixture of *Cornus officinalis* and *Achyranthes japonica* In Vitro and In Vivo

**DOI:** 10.3390/plants9091114

**Published:** 2020-08-28

**Authors:** Eunkuk Park, Chang Gun Lee, Jeonghyun Kim, Eunguk Lim, Seokjin Hwang, Seung Hee Yun, Yoonjoong Yong, Hyesoo Jeong, Ji Ae Kim, Hyun-Seok Jin, Seon-Yong Jeong

**Affiliations:** 1 Department of Medical Genetics, Ajou University School of Medicine, Suwon 16499, Korea; jude0815@hotmail.com (E.P.); dangsunsang@naver.com (C.G.L.); danbi37kjh@hanmail.net (J.K.); eunguk@ajou.ac.kr (E.L.); tjrwlshh@naver.com (S.H.); yun41101@ajou.ac.kr (S.H.Y.); 2 Department of Biomedical Sciences, Ajou University Graduate School of Medicine, Suwon 16499, Korea; 3 Nine B Company, Daejeon 34121, Korea; yoonjoong9b@gmail.com (Y.Y.); jhyesoo921@gmail.com (H.J.); ji.ae.kim@daum.net (J.A.K.); 4 Department of Biomedical Laboratory Science, College of Life and Health Sciences, Hoseo University, Asan 31499, Korea

**Keywords:** osteoporosis, *Cornus officinalis*, *Achyranthes japonica*, ovariectomized (OVX) mice, herbal medicine

## Abstract

Osteoporosis is a porous bone disease caused by bone density loss, which increases the risk of fractures. *Cornus officinalis* (CO) and *Achyranthes japonica* (AJ) have been used as traditional herbal medicine for various disorders in East Asia. Although the anti-osteoporotic effects of single extract of CO and AJ have already been reported, the synergistic effect of a combined mixture has not been studied. In this study, we investigated the effects of a CO and AJ herbal mixture on osteoporosis in in vitro and in vivo models. The results demonstrate that treatment with the CO and AJ mixture significantly promoted osteoblast differentiation of MC3T3-E1 mouse preosteoblasts through the upregulation of osteoblastic differentiation-associated genes such as alkaline phosphatase (*Alpl*), runt-related transcription factor 2 (*Runx2*), and bone gamma-carboxyglutamic acid-containing protein (*Bglap*)*,* while the mixture significantly inhibited differentiation of osteoclasts isolated from primary-cultured mouse monocytes. In addition, oral administration of CO and AJ mixture significantly prevented bone mineral density loss and trabecular bone structures in an ovariectomy-induced osteoporotic mouse model. These results suggest that the combination treatment of CO and AJ mixture might be a beneficial therapy for osteoporosis.

## 1. Introduction

Bone is a specialized connective tissue which protects and supports various organs of the body [1]. The skeleton is involved in body homeostasis by providing structure and supporting organs, storing minerals, and producing blood cells [2]. Bone remodeling occurs through two different processes: newly created bone formation (osteoblast) and resorptive old or damaged bone (osteoclast) [3]. The homeostasis between bone formation and resorption plays a critical role in the control of bone remodeling via replacing deteriorated old bone with new bone [4]. However, imbalanced bone homeostasis caused by hormones and inflammation triggers bone disorders, resulting in bone metabolic disorder, including osteoporosis [5]. Osteoporosis is an abnormal bone remodeling disease characterized by low quality bone mass and structural bone deterioration, resulting in bone fragility with enhanced vulnerability to fracture [5]. Osteoporosis mostly occurs in elderly women; 30% of women older than fifty years are diagnosed with osteoporosis during the menopausal period, due to the dysregulation of sex hormones that induce bone remodeling disorders [6]. A study reported that increased bone resorption by osteoclasts promotes bone mineral density (BMD) loss, leading to a high risk of the incidence of osteoporosis and bone fracture [7]. Although several medications such as denosumab, bisphosphonates, and raloxifene have been recommended for osteoporosis treatment, inappropriate use of chemical agents has a significant risk of adverse effects and increased cost [8,9,10].

In recent years, natural product-based therapies have emerged as alternative strategies for the treatment of various diseases, as they have fewer side effects [11]. Natural products contain numerous bioactive constituents for their therapeutic effects through multiple pathways, which correspond with the treatment for multi-factorial pathogenesis [12]. In addition, combination therapy of two or more natural products enhances multiple mechanisms of action and presents synergistic effects with fewer side effects than single treatment [13,14]. *Cornus officinalis* (CO) and *Achyranthes japonica* (AJ) have traditionally been used as herbal medicines in East Asia for their anti-oxidative, anti-inflammatory, and anti-cancer effects [15,16,17]. Previous studies have reported that CO inhibited osteoclast differentiation and promoted osteoblast differentiation, and AJ exhibited osteoprotective effects in ovariectomized (OVX) mice [18,19,20,21]. Although the individual anti-osteoporotic effects of *Cornus officinalis* (CO) and *Achyranthes japonica* (AJ) have been demonstrated, the synergistic effects of combined CO and AJ on osteoporosis have not been reported.

Therefore, this study examined the synergistic effects of combined therapy with CO and AJ on osteoporosis in vitro and in vivo. Our findings suggest that combined treatment of CO and AJ therapy could be an effective anti-osteoporotic treatment strategy.

## 2. Results and Discussion

### 2.1. CO and AJ Mixtures Induced an Increase in Osteoblast Differentiation Compared to Individual Treatment

**Figure 1:**
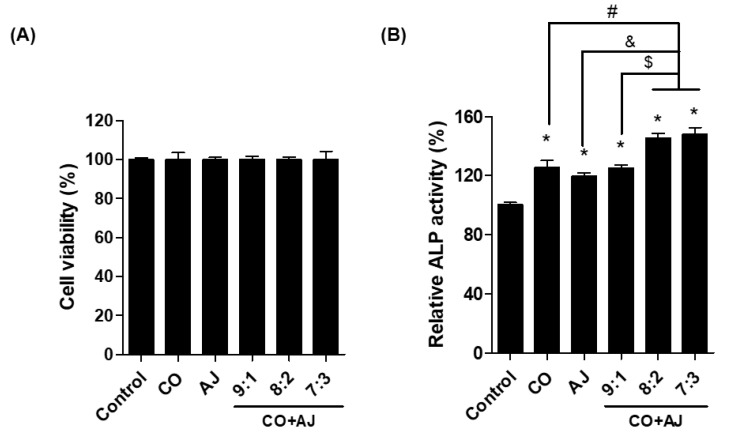
The effects of *Cornus officinalis* (CO) and *Achyranthes japonica* (AJ) mixture (CO+AJ) on osteoblast differentiation in MC3T3-E1 cells. Preosteoblast cells were cultured for 3 days with either CO or AJ or their combination at three different ratios (9:1, 8:2, and 7:3). (**A**) Relative cell viability was measured by the water-soluble tetrazolium salt (WST) assay. (**B**) Alkaline phosphatase (ALP) activity was accessed by adding 5 mM *p*-nitrophenylphosphate and the cleaved ALP substrate was detected at an absorbance of 405 nm. * *p* < 0.05 vs. Con, ^#^ *p* < 0.05 vs. CO, ^&^ *p* < 0.05 vs. AJ, ^$^ *p* < 0.05 vs. 9:1. (Tukey’s honest significant difference post hoc test, analysis of variance).

Osteoblast differentiation plays an essential role in the control of bone formation that reduces the pathogenesis of osteoporosis [22]. During osteoblast differentiation, alkaline phosphatase (ALP) is a biomarker for progressive osteoblast differentiation [23,24]. To examine the effect of the CO and AJ mixture on osteoblast differentiation, we evaluated the ALP activity after CO and AJ mixture treatment, compared to single treatment in pre-osteoblast MC3T3-E1 cells. Differentiation of mouse preosteoblast cells was induced for 3 days by treatment with β-glycerophosphate (10 mM) and ascorbic acid (50 μg/mL) and co-incubated with CO and AJ (10 μg/mL) at various ratios of CO and AJ mixture (9:1, 8:2, and 7:3). As a result, all conditions of either CO or AJ alone or CO and AJ mixture did not change the cell viability of MC3T3-E1 preosteoblasts (Figure 1A and Supplementary Figure S1). In addition, single treatment with CO and AJ (2, 10, and 50 μg/mL) promoted osteoblast activity (Supplementary Figure S2). However, treatment with the CO and AJ mixture increased ALP activity compared to single treatment, and the 7:3 ratio presented the highest ALP activity compared to other conditions (Figure 1B). Therefore, we used a herbal mixture of CO and AJ (10 μg/mL) at a 7:3 ratio for further experiments.

**Figure 2:**
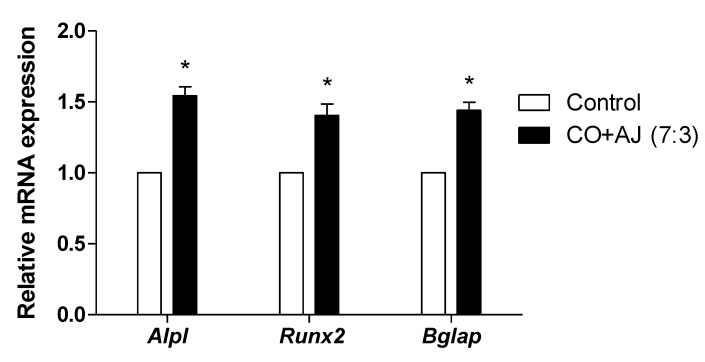
Effects of CO and AJ mixture (CO + AJ) on the mRNA expression levels of osteoblast differentiation-associated genes in preosteoblastic cells. MC3T3-E1 cells were incubated with a 7:3 ratio of CO and AJ mixture for 3 days and the mRNA expression levels of *Alpl*, *Runx2*, and *Bglap* were analyzed by qRT-PCR and subsequently normalized by mouse *Gapdh*. * *p* < 0.05 vs. Control.

Next, we confirmed the effect of CO and AJ mixture on osteoblast differentiation by evaluating mRNA expression levels of osteoblast differentiation-associated biomarkers such as *Alpl* (alkaline phosphatase, ALP), *Runx2* (runt-related transcription factor 2, RUNX2), and *Bglap* (bone gamma-carboxyglutamic acid-producing protein, osteocalcin). ALP is detected at the beginning of osteoblast differentiation and is considered to be a predictor of BMD in postmenopausal women [25]. RUNX2 modulates bone matrix gene expression during osteoblast differentiation, and genetic ablation of *Runx2* mice showed decreased osteoblast differentiation, resulting in a decrease in bone formation. [26,27]. Osteocalcin is secreted from osteoblast cells and promotes bone formation and calcium homeostasis [28]. To examine the mRNA expression levels of osteoblast differentiation-associated genes in MC3T3-E1 cells, preosteoblastic cells were treated with a 7:3 ratio of CO and AJ for 3 days, and the mRNA expression levels were assessed by quantitative reverse transcription polymerase chain reaction (qRT-PCR). As a result, the CO and AJ mixture enhanced the mRNA expression levels of *Alpl*, *Runx2,* and *Bglap,* compared to the control group (Figure 2). These results indicate that the CO and AJ mixture promoted osteoblast differentiation by upregulating mRNA expression levels of osteoblast-inducing genes.

### 2.2. CO and AJ Mixture Inhibited Osteoclast Differentiation Compared to Individual Treatment

**Figure 3:**
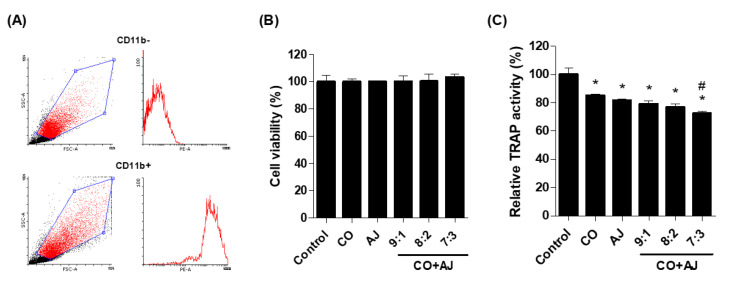
Effects of CO and AJ mixture on osteoclast activity. (**A**) Successfully isolated mouse monocytes were identified by phycoerythrin-conjugated CD11b antibody (monocyte-specific markers) using fluorescence-activated cell sorting analysis (FACS). Monocytes were induced by the addition of RANKL and M-CSF and treated with a 10 μg/mL mixture of CO and AJ at the indicated ratios (9:1, 8:2, and 7:3) for 6 days. (**B**) Cell viability of isolated monocytes was analyzed by the WST assay. (**C**) Tartrate-resistant acid phosphatase (TRAP) activity in differentiated osteoclasts was evaluated using an Acid-Phosphatase Leukocyte Kit. * *p* < 0.05 vs. Control, ^#^ *p* < 0.05 vs. CO. (Tukey’s honest significant difference post hoc test, analysis of variance).

Osteoclast differentiation is not only involved in the maintenance of bone metabolism and homeostasis but induces increased bone resorption associated with osteoporosis [29]. Osteoclasts are differentiated by macrophages derived from monocytes and stimulated by two main factors such as receptor activation of nuclear factor kappa B ligand (RANKL) and monocyte colony-stimulating factor (M-CSF) [30]. During osteoclast differentiation, tartrate-resistant acid phosphatase (TRAP) is a well-known specific marker that leads to the development and migration of mature osteoclasts [31]. Therefore, this study investigated the effect of CO and AJ mixture on osteoclast differentiation by measuring TRAP activity. Primary-cultured mouse monocytes isolated from femoral bones were identified by CD11b antibody (monocyte-specific surface markers), using fluorescence-activated cell sorting (FACS) (Figure 3A). Isolated cells were incubated with RANKL and M-CSF for osteoclast differentiation and co-treated with single or three different ratios of CO and AJ mixtures (9:1, 8:2, and 7:3) for 6 days. As a result, both CO and AJ (10 and 50 μg/mL) extract in our study did not affect cellular proliferation (Supplementary Figure S3), but inhibited TRAP activity (Supplementary Figure S4), as previously described in the literature [18,32]. In addition, mixtures of CO and AJ did not show toxic effect to osteoclasts during the differentiation period (Figure 3B). However, all ratios (9:1, 8:2, and 7:3) of CO and AJ mixture inhibited TRAP activity, and the CO and AJ mixture treatment at a 7:3 ratio produced the most preventive effects on osteoclast differentiation, compared to other treatments (Figure 3C). These results indicate that 7:3 mixture of CO and AJ exhibited a synergistic inhibitory effect on osteoclast differentiation.

### 2.3. CO and AJ Mixture Showed an Anti-Osteoporotic Effect on OVX-Induced Osteoporosis in Mice

**Figure 4:**
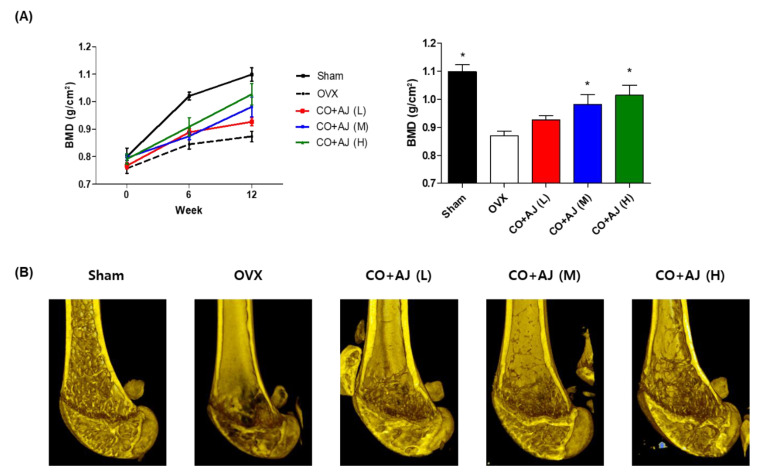
Effects of CO and AJ mixture on ovariectomy (OVX)-induced osteoporosis model mice. OVX mice was orally administrated a 7:3 ratio of the CO and AJ mixture with CO+AJ (L) (75 mg/kg/day), CO+AJ (M) (150 mg/kg/day), and CO+AJ (H) (300 mg/kg/day) concentrations for 12 weeks. (**A**) Bone mineral density (BMD) was analyzed at 0, 6, and 12 weeks, using a PIXI-mus bone densitometer. * *p* < 0.05 vs. OVX (Tukey’s honest significant difference post hoc test, analysis of variance). (**B**) Transverse micro-CT images were obtained at the last day of the animal experiment.

Ovariectomized mice are an experimental model for investigating postmenopausal osteoporosis because hormone deficiency promotes the loss of bone mass and BMD [33]. Based on results of osteoblasts and osteoclasts study, the anti-osteoporotic effect of CO and AJ mixture on OVX mice were evaluated. OVX mice were orally administered for 12 weeks at a 7:3 ratio of CO and AJ mixture (75, 150, and 300 mg/kg/day). During the animal experiment, the BMD of the right femur was evaluated using a PIXI-mus bone densitometer at 0, 6, and 12 weeks. At the last day of the animal experiment, transverse micro-computed tomography (micro-CT) images of the right femur were scanned. As a result, OVX mice showed significantly reduced BMD and osteoporotic trabecular bone structure loss, compared to the sham positive control group. Nevertheless, OVX-induced loss of BMD and trabecular bone structure was prevented by CO and AJ mixture administration at 150 and 300 mg/kg/day (Figure 4). In addition, non-surgery mice treated with the CO and AJ mixture (75, 150 and 300 mg/kg/day) did not present any in vivo toxic effects, including total body weight change, for 12 weeks of administration (Supplementary Figure S5), and food intake (data not shown) differences between sham and CO+AJ treated groups. Taken together, the results indicate that the treatment with the CO and AJ mixture inhibited OVX-induced osteoporosis in mouse model.

## 3. Materials and Methods

### 3.1. Extraction and Sample Preparation

Air-dried roots of *Achyranthes japonica* (AJ) were purchased Dongwoodang Pharmacy Co., Ltd. (Yeongcheon, Gyeongsangbuk-do, Korea). One hundred grams of AJ roots were extracted with 1 L of a 70% ethanol solution or 60 °C for 8 h. Air-dried fruit of *Cornus officinalis* (CO) were obtained from the farm at Icheon and Yangpyeong (Gyeonggi-do, Korea). One hundred grams of CO fruit were extracted with 1 L of a 70% ethanol solution at 60 °C for 8 h. The AJ and CO extract were filtered with filter paper (CHMLAB, Terrassa, Barcelona, Spain). After filtration, the methanol extracts of CO and AJ were evaporated using a rotary vacuum evaporator (EYELA, Tokyo, Japan) at 45 °C. Concentrated AJ and CO extract were dried in a freeze dryer (IiShin BioBase Co., Ltd., Dongducheon, Gyeonggi-do, Korea) at −70 °C for 72 h. Lyophilized CO and AJ extract were stored at −20 °C. The powder of CO or AJ extract was dissolved in distilled water and filtered through a 0.45 μm PVDF syringe filter (Microlab Scientific, Mongkok, Hong Kong) before the experiments.

### 3.2. Osteoblast and Osteoclast Differentiation

The Preosteoblast cell line (MC3T3-E1 subclone 4) was purchased from ATCC (American Type Culture Collection) (ATCC® CRL-2593™) and cultured in α-modified minimal essential medium (α-MEM) containing streptomycin (100 μg/mL), 10% fetal bovine serum, and penicillin (100 U/mL). Single CO or AJ extracts or various ratios of CO and AJ mixtures were diluted in cell culture medium. To induce osteoblast differentiation, MC3T3-E1 cells were incubated with β-glycerophosphate (10 mM) and ascorbic acid (50 μg/mL) for 3 days. Primary-cultured mouse monocyte cells were isolated from mouse bone marrows according to the guidelines of the Institutional Animal Care and Use Committee of Ajou University School of Medicine (2016-0062). Isolated monocyte cells were confirmed by FACS using Aria III cell sorter and Diva software (BD Biosciences, San Jose, CA, USA). Monocytes were incubated with α-MEM containing 50 ng/mL of RANKL (PeproTech, Cranbury, NJ, USA), and 30 ng/mL M-CSF (PeproTech, Rocky Hill, CT, USA) for osteoclast differentiation. MC3T3-E1 and monocytes were cultured in a humidified atmosphere at 5% CO_2_ and 37 °C.

### 3.3. Measurement of ALP and TRAP Activity

Preosteoblast cells were lysed in a buffer containing 10 mmol/L Mg^2+^, 1 mM Tris-HCl (pH 8.8) 0.5% and Triton X-100 at 4 °C. Cell lysates were incubated with 5 mM *p*-nitrophenylphosphate as an ALP substrate. ALP activity was measured the optical density at 405 nM (BIO-RAD, Hercules, CA, USA). The osteoclast differentiation was evaluated by TRAP activity using an Acid-Phosphatase Kit (Sigma-Aldrich, St. Louis, MO, USA).

### 3.4. Cell Viability

The cells were incubated in 96-well plates for 24 h and treated with single or different ratios of CO and AJ mixtures in the culture medium for either 3 d (MC3T3-E1 cells) or 6 d (monocytes). The water-soluble tetrazolium salt (WST) assay for cell viability was assessed using the Cell Viability Assay Kit of EZ-Cytox (Daeil, Seoul, Korea). Cells were cultured in 20 μL WST solution for 4 h and cell viability was accessed by a microplate reader at the absorbance values at 450 nm (BioTek, Winooski, VT, USA).

### 3.5. Quantitative Reverse-Transcription PCR (qRT-PCR)

Total RNA of cultured cells was extracted using TRIzol reagent (Invitrogen, Carlsbad, CA, USA) and RNA quality was confirmed by the optical density at 260/280 nM. Complementary DNA (cDNA) was synthesized with oligo (dT) 12-18 primer, using Synthesis Kit of RevertAid™ H Minus First Strand cDNA (Fermentas, Hanover, NH, USA). Expression levels were evaluated by quantitative reverse-transcription polymerase chain reaction (qRT-PCR), using the ABI Prism 7000 Sequence Detection System (Applied Biosystems, Foster City, CA, USA). All polymerase chain reactions (total volume 10 μL containing 100 ng of cDNA) were performed using a qPCR Kit of SYBR Green I (TaKaRa, Shiga, Japan). The specific qRT-PCR primers for the expression levels of osteoblast differentiation-associated biomarkers were as follows: 5′-CCT CAG TGA TTT AGG GCG CA-3′ and 5′-TAA AGT GAC AGT GGA CGG TCC C-3′ for mouse *Runx2,* 5′-GGC CAT CCT ATA TGG TAA CGG G-3′ and 5′-TCC CAC GTT TTC ACA TTC GG-3′ for mouse *Alpl*, 5′-ATG GCT TGA AGA CCG CCT ACA-3′ and 5′-TAG TGA ACA GAC TCC GGC GCT A-3′ for mouse *Bglap*, and 5′-GAC GGA CAC ATT GGG GGT AG-3′ and 5′-CCT AGC CTC ATA CCC CCA G-3′ for mouse *Gapdh*. The relative mRNA expression levels were normalized by mouse *Gapdh*. Results were presented as 2^-ΔΔCt^ (ΔΔCt = ΔCt_treatment_ - Ct_control_) and fold change was analyzed by comparing with the untreated control group.

### 3.6. Ovariectomized Osteoporotic Model Mice Experiment

Sham-operated and OVX 8-week-old female ddY mice (*n* = 35) were obtained from Shizuoka Laboratory Center Inc. (Hamamatsu, Japan). The mice were housed on a 15 mL/day tap water and a 5.0 g/d Formula-M07 (Feedlab Co. Ltd., Hanam, Korea) and. All mice were maintained in clear plastic cages (less than 5 in a cage) at 23 ± 2 °C, illumination (12-h light/dark cycle) and humidity (55 ± 5%) Mice were orally administered by gavage for 12 weeks at a 7:3 ratio of CO and AJ mixture with CO + AJ (L) (75 mg/kg/day), CO + AJ (M) (150 mg/kg/day), and CO + AJ (H) (300 mg/kg/day) concentrations (*n* = 7 in each group). The measurement of BMD was accessed at three points (0, 6, and 12 weeks). Animal experiments were accepted and conducted according to the guidelines of the Institutional Animal Care and Use Committee of Ajou University School of Medicine (AMC-133).

### 3.7. Analysis of Bone Marrow Density (BMD) and Transverse Micro-CT Imaging in Bone Tissues

Mice were anesthetized intraperitoneally by injection of zolazepam/tiletamine (Zoletil; Virbac Laboratories, Carros, France) and fixed on the specimen tray for measurement. The BMD was detected by a PIXI-mus bone densitometer and analyzed with on-board PIXI-mus software (GE Lunar, Madison, WI, USA). The right femur was removed and transverse micro-CT images were scanned under condition of a current of 400 μA, a voltage of 60 kV, a charge-coupled device (CCD) camera readout of 1280 × 1280, an exposure time of 400 ms, and rotation steps of 360°, using a high-energy spiral scan micro-CT, Skyscan 1173 (Bruker, Kontich, Belgium). Reconstruction of two- and three-dimensional axial images was completed by the NRecon software (Bruker microCT, North Billerica, MA, USA) and displayed with a spatial resolution of 8.88 μM by an Inveon Research Workplace (Siemens). For the improvement of slice-by-slice manual tracing of the trabecular bone shapes, axial reformats were conducted.

### 3.8. Statistical Analysis

All experiments were performed in triplicate, and all statistical analyses were accessed by the software package of SPSS 11.0 for Windows (SPSS Inc. Chicago, IL, USA). The results of the bar graphs are represented as the mean ± standard error of the mean (SEM). Statistically significant differences between groups were analyzed by Student’s *t*-test. For the comparisons of multiple groups, a one-way analysis of variance (ANOVA) was performed, followed by Tukey’s honest significant difference (HSD) post hoc test. A probability value less than 0.05 (*p* < 0.05) was considered statistically significant.

## 4. Conclusions

This study investigated the synergistic anti-osteoporosis effects of CO and AJ mixture on MC3T3-E1 (osteoblasts) and monocytes (osteoclasts) in vitro and an OVX-induced osteoporotic model in vivo. The combination of CO and AJ at a 7:3 ratio increased osteoblast differentiation through the up-regulation of osteoblast-promoting genes (*Alpl*, *Runx2,* and *Bglap*), whereas the osteoclast activity was reduced by treatment with CO and AJ mixture. Oral administration of CO and AJ prevented OVX-induced bone loss in osteoporotic animal model, by inhibiting the loss of BMD and trabecular bone structural properties. Our results suggest that the combined treatment of CO and AJ may be a potent therapeutic agent against osteoporosis.

## Supplementary Materials

The following are available online at https://www.mdpi.com/2223-7747/9/9/1114/s1, Figure S1: Effects of individual CO and AJ extract on cell proliferation in preosteoblast MC3T3-E1 cells. Cells were treated with ascorbic acid (50 μg/ml) and β-glycerophosphate (10 mM) and cultured with three different concentrations (2, 10 and 50 μg/ml) of either CO or AJ and cell proliferation was assessed by WST, Figure S2: Effect of individual CO and AJ extract on alkaline phosphatase (ALP) activity in the osteoblast-lineage cell lines. Control: non-treated cells, * *p* < 0.05 vs. Control, ^#^ *p* < 0.05 vs. CO2 or AJ2. (Tukey’s honest significant difference post hoc test, analysis of variance), Figure S3: Effects of single CO and AJ extract on cell proliferation in mouse monocytes from bone marrow. Cells were treated with either CO or AJ at two different concentrations (10 and 50 μg/ml) and cell proliferation was assessed by WST, Figure S4: Effects of single CO and AJ extract on tartrate-resistant acid phosphatase (TRAP) activity in mouse monocytes from bone marrow. Control: non-treated cells. *: *p*<0.05 vs. Control, Figure S5: Changes of total body weight for 12 weeks in non-surgery mice.
