# Supplementary material for: Anti-Osteoporotic Effects of the Herbal Mixture of Cornus officinalis and Achyranthes japonica In Vitro and In Vivo
Source: Plants (Basel). 2020 Aug 28;9(9):1114. doi: 10.3390/plants9091114 (PMC7570351; doi:10.3390/plants9091114)
Supplement: Supplementary file 1 [file plants-09-01114-s001.pdf]

Anti-osteoporotic effects of the extract mixture of *Cornus officinalis* and *Achyranthes japonica in vitro* and *in vivo*

Eunkuk Park, Chang Gun Lee, Jeonghyun Kim, Eunguk Lim, Seokjin Hwang,

Seung Hee Yun, Yoon-joong Yong, Ji Ae Kim, Hyun-Seok Jin and Seon-Yong Jeong

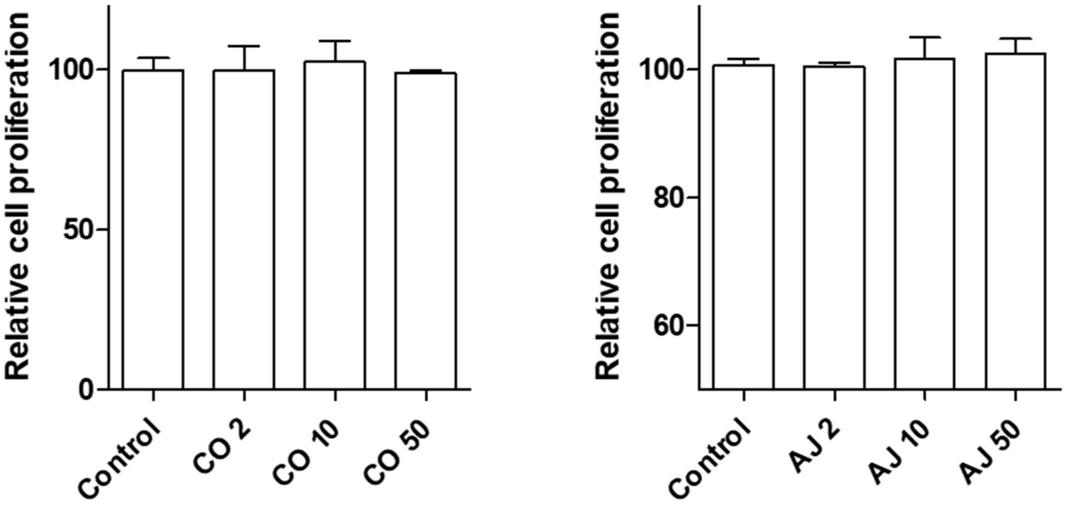

Supplementary Figure S1. Effects of individual CO and AJ extract on cell proliferation in preosteoblast MC3T3-E1 cells. Cells were treated with ascorbic acid (50 μg/ml) and β-glycerophosphate (10 mM) and cultured with three different concentrations (2, 10 and 50 μg/ml) of either CO or AJ and cell proliferation was assessed by WST.

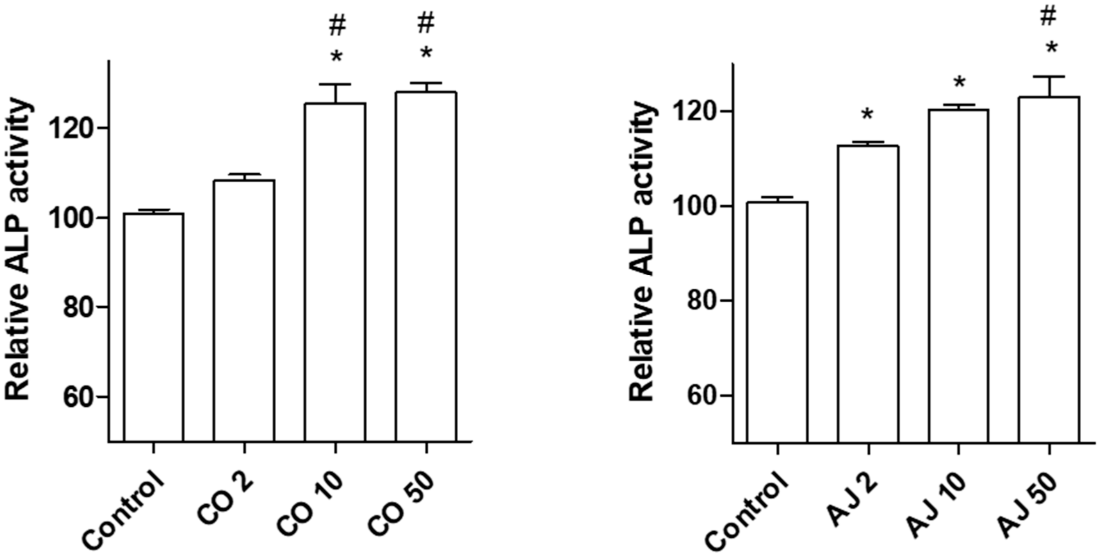

Supplementary Figure S2. Effect of individual CO and AJ extract on alkaline phosphatase (ALP) activity in the osteoblast-lineage cell lines. Control: non-treated cells, $^{*}p < 0.05$ vs. Control, $^{\#}p < 0.05$ vs. CO2 or AJ2. (Tukey's honest significant difference post hoc test, analysis of variance).

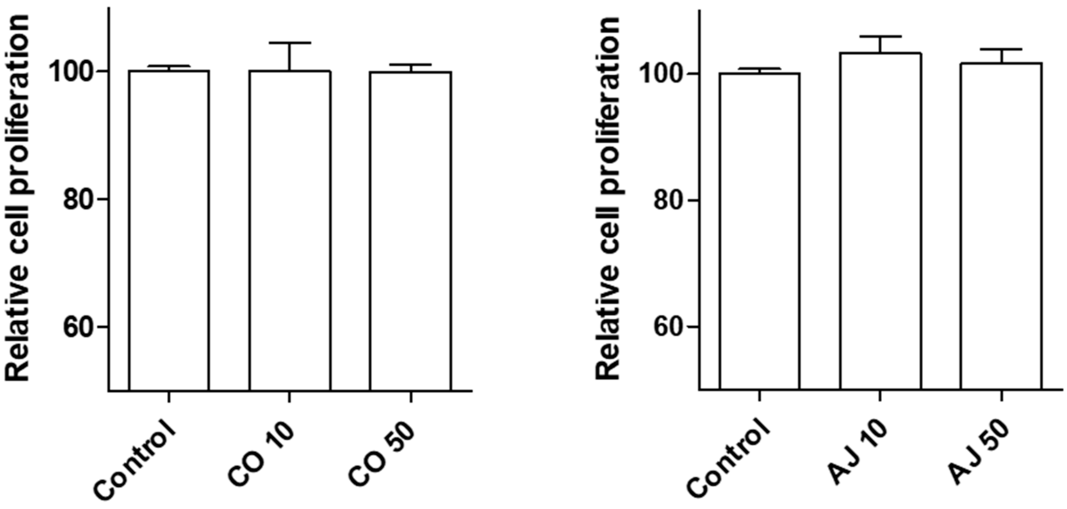

Supplementary Figure S3. Effects of single CO and AJ extract on cell proliferation in mouse monocytes from bone marrow. Cells were treated with either CO or AJ at two different concentrations (10 and 50 μg/ml) and cell proliferation was assessed by WST.

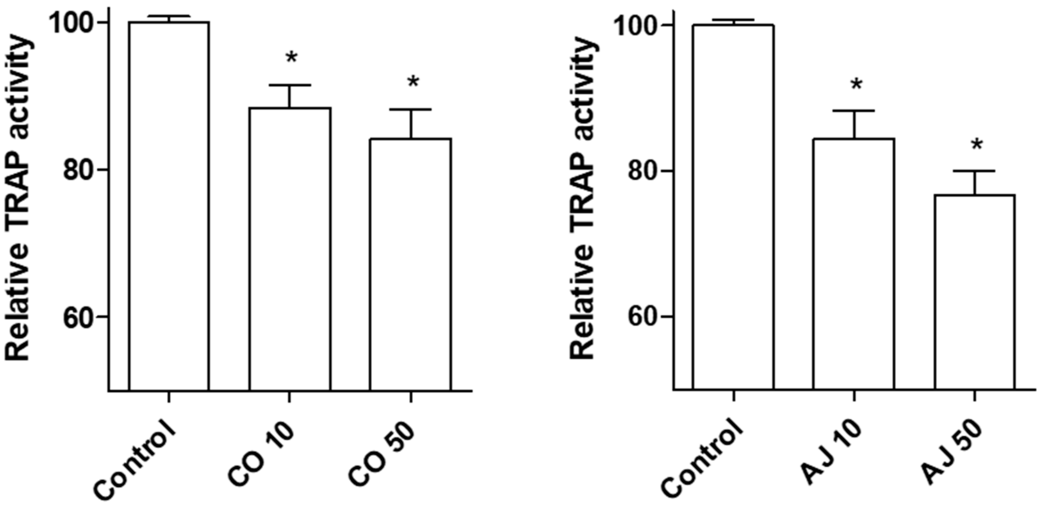

Supplementary Figure S4. Effects of single CO and AJ extract on tartrate-resistant acid phosphatase (TRAP) activity in mouse monocytes from bone marrow. Control: non-treated cells. *: $p<0.05$ vs. Control.

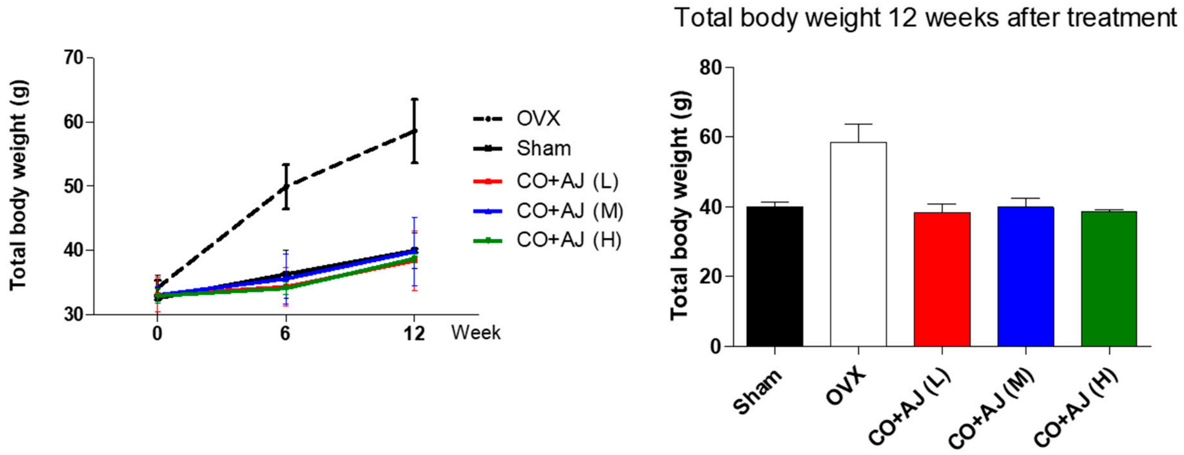

Supplementary Figure S5. Changes of total body weight for 12 weeks in non-surgery mice.
